# Debottlenecking the biological hydrogen production pathway of dark fermentation: insight into the impact of strain improvement

**DOI:** 10.1186/s12934-022-01893-3

**Published:** 2022-08-19

**Authors:** Yujin Cao, Hui Liu, Wei Liu, Jing Guo, Mo Xian

**Affiliations:** CAS Key Laboratory of Biobased Materials, Qingdao Institute of Bioenergy and Bioprocess Technology, Chinese Academy of Sciences, Qingdao, 266101 China

**Keywords:** Biological hydrogen production, Dark fermentation, Metabolic engineering, Strain improvement

## Abstract

Confronted with the exhaustion of the earth’s fossil fuel reservoirs, bio-based process to produce renewable energy is receiving significant interest. Hydrogen is considered as an attractive energy carrier that can replace fossil fuels in the future mainly due to its high energy content, recyclability and environment-friendly nature. Biological hydrogen production from renewable biomass or waste materials by dark fermentation is a promising alternative to conventional routes since it is energy-saving and reduces environmental pollution. However, the current yield and evolution rate of fermentative hydrogen production are still low. Strain improvement of the microorganisms employed for hydrogen production is required to make the process competitive with traditional production methods. The present review summarizes recent progresses on the screening for highly efficient hydrogen-producing strains using various strategies. As the metabolic pathways for fermentative hydrogen production have been largely resolved, it is now possible to engineer the hydrogen-producing strains by rational design. The hydrogen yields and production rates by different genetically modified microorganisms are discussed. The key limitations and challenges faced in present studies are also proposed. We hope that this review can provide useful information for scientists in the field of fermentative hydrogen production.

## Key points

- Hydrogen can be generated by microorganisms.
- Dark fermentation is efficient for biological hydrogen production.
- Strain improvement is critical to enhancing hydrogen-producing ability.

## Introduction

Energy plays a critical role in our daily lives and has a direct impact on economic, social and cultural activities. At present, the global energy demand mainly depends on fossil fuels, which account for over 85% of our energy supply [1]. Fossil fuels are probably finite and non-renewable energy resources. The decrease of fossil fuels reserves has led to the rising of their international prices. In addition, combustion of fossil fuels releases significant amounts of harmful gases and thus causes devastating impact on the environment and human health. The joint challenges of dwindling fossil fuel resources and anthropogenic climate change have prompted us to explore alternative energy sources. Many researchers have been working to explore new sustainable energy sources that could replace fossil fuels [2].

Among the various new fuel options, hydrogen has been identified as a non-polluting energy carrier because its combustion product is H_2_O without CO_2_ emissions. Hydrogen offers many beneficial features, such as being harmless to mammals and the environment. It is found to be one potential alternative to fossil fuel energy and has drawn a worldwide attention as a future energy source. Besides, hydrogen has a very high energy density on the mass basis. The major problem in the utilization of hydrogen as a fuel is its unavailability in nature and the need for inexpensive manufacturing methods. Hydrogen can be generated from natural gas, oil, coal gasification or electrolysis of water [3]. Currently, hydrogen production from fossil resources makes up the main proportion for hydrogen demand while electrolysis of water accounts for 4% of the global demand [4, 5]. However, hydrogen produced from fossil fuels is non-renewable. This promotes us to seek for a more sustainable source of hydrogen and the idea of fermentation hydrogen production is put forward. Compared with water electrolysis, fermentative hydrogen production has also received high support from the market and the government. Up to now, the electrolysis of water is more practical. However, electrolytic hydrogen is considerably expensive unless low-cost renewable electricity is available [6]. Although the efficiency of fermentative hydrogen production is lower than that of water electrolysis, it is a feasible solution for the treatment of organic wastes because it can achieve two goals simultaneously: reducing the environmental burden and producing clean energy.

## Dark fermentation—a promising method for hydrogen production

Biological hydrogen production is defined as hydrogen generated biologically from the cultivation of microorganisms. It is different from traditional hydrogen producing methods. Biological hydrogen production can be achieved by direct or indirect biophotolysis of water using algae and cyanobacteria, photo fermentation by photosynthetic bacteria [7] and dark fermentation by diverse groups of heterotrophic bacteria [8]. Biophotolysis and photo fermentation could utilize the widely available solar energy for the production of hydrogen. However, these methods show low light conversion efficiencies and require expensive hydrogen impermeable photo-bioreactors, hampering their further applications. Dark fermentation is an attractive option for hydrogen production. In comparison to the other two means of producing hydrogen, dark fermentation owns many unique excellent properties:

1. Dark fermentation does not require a direct input of light energy. It is capable of constantly producing hydrogen throughout the day and night [9].
2. The hydrogen evolution rate (the amount hydrogen produced per unit of time) of dark fermentation is higher compared with biophotolysis and photo fermentation processes [10].
3. Dark fermentation could utilize a wide spectrum of potential substrates, including renewable biomass and organic waste materials, which results in a relatively lower cost [11].
4. Dark fermentation could use already existing reactor and be easily scaled up through bioreactor design [12].

As discussed above, the excellent properties have given dark fermentation a bright prospect for hydrogen production. It is considered to be the most practical among the various biological hydrogen production methods at present. However, the current productivity and yield are still not economically feasible for industrial applications. This bioprocess must be improved through various strategies, among which, screening of more efficient microbial strains is an important aspect. In the past few years, state-of-the-art review articles have been published focusing on the feedstocks [13–15], inhibition factors [16], microbial communities [17], metabolic engineering [18–20], cell immobilization [21], bioinformatics approaches [22] and bioreactor design [23] aspects for hydrogen production by dark fermentation. A comprehensive review of strain improvement strategies to enhance fermentative hydrogen production is urgently required.

This review throws light on hydrogen-producing microorganisms used for dark fermentation. The classification and performances of these microorganisms are discussed in detail. Strain improvement by random mutagenesis and screening is employed to enhance their hydrogen-producing capability. Moreover, the metabolic pathways for fermentative hydrogen production are described and rational design strategies to engineer the hydrogen-producing strains are highlighted. Finally, challenges and future research trends in the field of fermentative hydrogen production are put forward. We hope that this review can give some implications to the industrialization of biological hydrogen production.

## Fermentative hydrogen-producing microorganisms

Microorganisms employed for biological hydrogen production by dark fermentation are mainly bacteria. Although several rumen fungal strains were reported to be able to produce hydrogen, their feasibility for fermentation was still not tested [24]. Up to now, a number of fermentative hydrogen-producing bacteria have been isolated and characterized to some extent. These microorganisms can be generally classified into three categories: strict anaerobes, facultative anaerobes and aerobes. Several representative species that have been extensively investigated are listed in Table 1.

**Table 1:** Various fermentative hydrogen producing bacteria and their maximum hydrogen yields reported

Type	Genus	Species	Hydrogen yields	References
Strict anaerobes	*Aphanothece*	*A. halophytica*	1864 nmol/mg DW^a^	[35]
	*Clostridium*	*C. acetobutylicum*	2.0 mol/mol glucose	[28]
		*C. beijerinckii*	2.31 mol/mol xylose	[29]
		*C. butyricum*	2.78 mol/mol sucrose	[31]
		*C. pasteurianum*	2.33 mol/ mol glucose	[119]
		*C. paraputrificum*	1.9 mol/mol N-acetyl-D-glucosamine	[120]
		*C. thermolacticum*	3.0 mol/mol lactose	[30]
		*C. tyrobutyricum*	3.7 mol/mol inulin-type sugar	[121]
		*C. perfringens*	4.68 mol/mol glucose	[122]
		*C. bifermentans*	3.29 mol/mol glucose	[122]
	*Caldicellulosiruptor*	*C. bescii*	86 mL/g wastewater biosolids	[39]
	*Ruminococcus*	*R. albus*	2.01 mol/mol glucose	[123]
		*R. flavefaciens*	0.59 mol/mol cellulose^b^	[34]
	*Thermotoga*	*T. maritima*	2.2 mol/mol glucose	[124]
	*Thermoanaerobacterium*	*T. thermosaccharolyticum*	10.86 mmol/g cellulose	[125]
	*Thermococcus*	*T. kodakaraensis*	24.9 mmol/(g DW·h)^a^	[126]
		*T. onnurineus*	3.13 mol/mol starch^b^	[127]
Facultative anaerobes	*Bacillus*	*B. anthracis*	2.42 mol/mol mannose	[128]
		*B. coagulans*	2.28 mol/mol glucose	[129]
		*B. cereus*	1.15 mol/mol glucose	[130]
		*B. thuringiensis*	1.67 mol/mol glucose	[131]
	*Citrobacter*	*C. amalonaticus*	1.24 mol/mol glucose	[44]
		*C. freundii*	0.83 mol/mol glucose	[45]
		*C. intermedius*	1.1 mol/mol glucose	[46]
	*Enterobacter*	*E. aerogenes*	2.23 mol/mol glucose	[132]
		*E. cloacae*	2.2 mol/mol glucose	[133]
	*Enterococcus*	*E. faecium*	1.69 mol/mol glucose	[134]
	*Escherichia*	*E. coli*	2 mol/mol glucose	[43]
	*Klebsiella*	*K. oxytoca*	9.33 mmol/g sucrose	[135]
		*K. pneumoniae*	2.07 mol/mol glucose	[47]
Aerobes	*Alcaligenes*	*A. eutrophus*	100 μmol/(gProtein·h)	[52]

^a^DW, dry weight

^b^The amount of cellulose and starch was given as glucose equivalents

### Strict anaerobes

Among a large variety of microorganisms capable of producing hydrogen, strict anaerobes are the most widely studied. Strict anaerobes do not carry out oxidative phosphorylation and mainly generate ATP by substrate level phosphorylation and flavin-based electron-bifurcation process during fermentation. These microorganisms are incapable of growing in the presence of oxygen. The currently isolated strict anaerobes for hydrogen production include *Clostridia*, rumen bacteria, methanogenic bacteria and thermophilic archaea. The Gram-positive bacteria of the *Clostridium* genus are the dominant microorganisms in anaerobic communities for hydrogen production [25–27]. They can naturally produce hydrogen at a high rate by mixed acid fermentation. In addition, most of the *Clostridia* could form spores, which make the bacteria easy to handle in industrial application. The dominant culture of *Clostridia* can be easily obtained by heat treatment. *Clostridia* can form spores that can withstand high temperature and survive. Spores of *Clostridia* are generated at high temperature, while most miscellaneous bacteria were killed under such conditions. When suitable conditions are provided, the spores formed at high temperatures can be activated for hydrogen production. *Clostridium* species could also be cultured under fermenter-controlled conditions. They produce hydrogen during the logarithmic stage and then shift to solvent production in the stationary stage. *Clostridia* could utilize a variety of substrates for hydrogen production, including glucose [28], xylose [29], lactose [30], sucrose [31]. The hydrogen yields of these strains were relatively high (> 2 mol/mol substrates). Recently, the whole genome of the representative of this genus, *C. acetobutylicum*, has been completely sequenced [32] and genome analyses of this genus have been much more advanced since then, leading to a more thorough understanding for its hydrogen production mechanisms.

Rumen bacteria, such as *Ruminococcus albus*, are also good candidates for hydrogen production. These microorganisms are capable of hydrolyzing cellulose, showing a potential to directly utilize the widespread plant biomass as the substrate [33]. But the hydrogen yield (0.59 mol/mol cellulose, calculated as glucose equivalents) was much lower to ferment cellulose than glucose [34]. The unicellular cyanobacterium *Aphanothece halophytica* was a potential hydrogen-producing strain. It was able to grow in natural seawater under anoxic conditions for long-term production (up to 14 days) [35]. Many anaerobic thermophilic bacteria have demonstrated the ability to produce hydrogen and these bacteria are general thought to belong to archaea. Thermophilic hydrogen production at high temperatures processes the advantages of high gas production rate, reduced consumption of cooling water, less chance of feedstock contamination and enhanced mass transfer efficiency [36].

In general, strict anaerobes represent the largest category of hydrogen producing microorganisms. However, the highest hydrogen yield achieved by strict anaerobes was lower than 3 mol/mol glucose reported in the literature [37]. It should be agreed that strict anaerobes have the potential for higher hydrogen production yields. Strict anaerobes can only grow in the absence of oxygen. The hydrogen element in the substrate is unable to generate water through aerobic fermentation so as to obtain a higher yield. In addition, there is another pathway for strict anaerobes to produce hydrogen from NADH through NADH: ferredoxin oxidoreductase [38]. Most strict anaerobes can grow in simple media containing different organic compounds. More specifically, many species of them could directly utilize lignocellulosic biomass and waste organic compounds [39] as the substrate for hydrogen evolution, which might greatly decrease the production cost of bio-hydrogen.

### Facultative anaerobes

Biological hydrogen production could also be achieved by many species of facultative anaerobic bacteria. Facultative anaerobes can grow in both aerobic and anaerobic environments. Whereas oxygen kills strict anaerobes, facultative anaerobes are less sensitive to oxygen. They usually grow very quickly under aerobic conditions and transform from aerobic to anaerobic metabolism as oxygen becomes depleted. As a consequence, facultative anaerobes are considered better than strict anaerobes because they can be cultured to a high cell density with oxygen and then produce hydrogen at a high rate when the oxygen supply is stopped [40]. Even for facultative anaerobes, the presence of oxygen also leads to electron transfer to oxygen and inhibits hydrogen generation. However, the activities of enzymes involved in hydrogen production recover rapidly when oxygen is removed.

The enteric bacteria, such as *Enterobacter* sp., *Escherichia coli* and *Citrobacter* sp. are the best characterized facultative anaerobes for their hydrogen-producing abilities. The model bacterium *E. coli* has classically been utilized as a cell factory for the production of recombinant proteins and various value-added metabolites [41]. The production of hydrogen from diverse carbohydrates by *E. coli* has been demonstrated by many previous researchers. Considering its clear genetic background and ease for DNA manipulation, metabolic engineering strategies have been employed for *E. coli* to enhance its hydrogen-producing ability [42]. Mutant *E. coli* strains showed the highest yield of about 2 mol/mol glucose cultured at nutrient limitation conditions [43]. Compared with *E. coli*, many species of *Enterobacter* could utilize much broader substrates and thus enlarged their application range. Three species of *Citrobacter* including *C. amalonaticus*, *C. freundii* and *C. intermedius* were also investigated for hydrogen production. The maximum hydrogen yield merely reached 1.24 mol/mol glucose [44–46]. All of these enteric bacteria are mesophilic and have an optimal growth temperature of around 37 °C. Therefore, these bacteria are easy to be controlled and cultured in the fermenter, which can be scaled up to an industrial level. *Klebsiella* sp. was also a good candidate for hydrogen production due to its rapid growth rate and ability to produce various valuable by-products. The volumetric productivity and yield of hydrogen of *K. pneumoniae* reached 482 ml/(L·h) and 2.07 mol/mol glucose, respectively [47]. The theoretical hydrogen yields of facultative anaerobes were lower than that of strict anaerobes. One exception is that *Enterobacter cloacae* was able to enhance its hydrogen yield up to 3.9 mol/mol glucose, which should be due to decreased partial pressure of hydrogen [48]. In addition, facultative anaerobes could be used to consume oxygen and maintain anaerobic conditions for hydrogen production by strict anaerobes [49].

### Aerobes

Most of the known hydrogenases have a high sensitivity to oxygen. In the presence of oxygen, these enzymes do not show any activity especially to hydrogen production. Therefore, aerobes are traditionally considered to be incapable of producing hydrogen. The only example of hydrogen evolution by aerobic bacteria was reported in diazotroph bacteria, such as *Azotobacter vinelandii* [50, 51]. Aerobic hydrogen production by the aerobe *Alcaligenes eutrophus* could be also achieved by shifting the bacterial culture to anaerobic conditions [52]. Aerobic hydrogen production is not preferable since the hydrogen generated would be taken by the off-gas, thus hampering the separation and collection processes. However, the studies on hydrogenases resistant to oxygen and other inactivating factors would give some implication to the current hydrogen-producing systems.

**Fig. 1:**
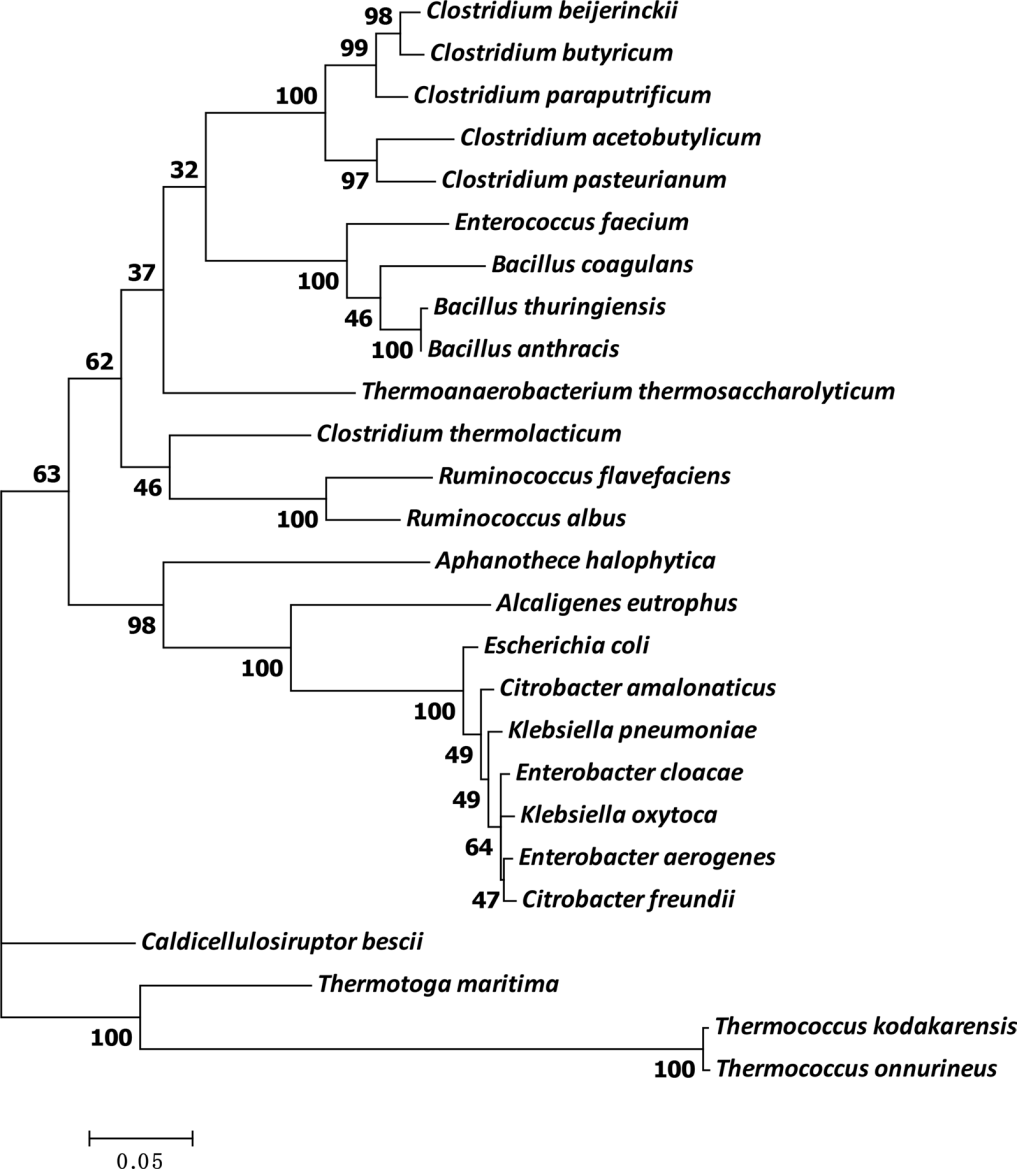
Maximum parsimony tree showing phylogenetic relationships of different fermentative hydrogen-producing microorganisms inferred from 16S rDNA gene sequences. Phylogenetic analysis was conducted using the MEGA7 software. Sequence alignment was performed by ClustralW. The phylogenetic tree was constructed using the Neighbor-Joining method. GenBank accession numbers for the 16S or 18S rDNA sequences are: *A. eutrophus*, LN995683; *A. halophytica*, AJ000708; *B. anthracis*, MK066928; *B. coagulans*, DQ347840; *B. thuringiensis*, EF206345; *C. acetobutylicum*, NR_113246; *C. amalonaticus*, NR_104823; *C. beijerinckii*, AB678386; *C. bescii*, NR_074788; *C. butyricum*, LN828942; *C. freundii*, DQ517286; *C. pasteurianum*, EF140981; *C. paraputrificum*, NR_113021; *C. thermolacticum*, NR_026113; *E. aerogenes*, NR_114737; *E. cloacae*, DQ202394; *E. coli*, J01859; *E. faecium*, NR_112039; *K. oxytoca*, NR_119277; *K. pneumoniae*, KC249934; *R. albus*, NR_113032; *R. flavefaciens*, AF104841; *T. kodakarensis*, NR_028216; *T. maritima*, AJ401021; *T. onnurineus*, NR_074373; *T. thermosaccharolyticum*, KX462132

The taxonomic status, hydrogen production and substrates utilization ability of these microorganisms were quite different. Phylogenetic analysis of these strains [53] based on the 16S rDNA sequence is shown in Fig. 1. These species could be divided into two distinct groups, one consisting of *Thermococcus kodakaraensis* and *Thermococcus onnurineus* while the other consisting of all the other bacteria. Members of the genus *Thermococcus* are all archaea. They have a distant relationship to eubacteria. The eubacteria could be further classified into several subfamilies according to their genera and species. There seems to be no direct correlation between the taxonomic status and their hydrogen-producing capability. The genetic relationship of the hydrogen-producing microorganisms depends on the taxonomy instead of their oxygen requirement. Their ability to produce hydrogen should be the result of convergent evolution.

## Metabolic pathways of biological hydrogen production by dark fermentation

**Fig. 2:**
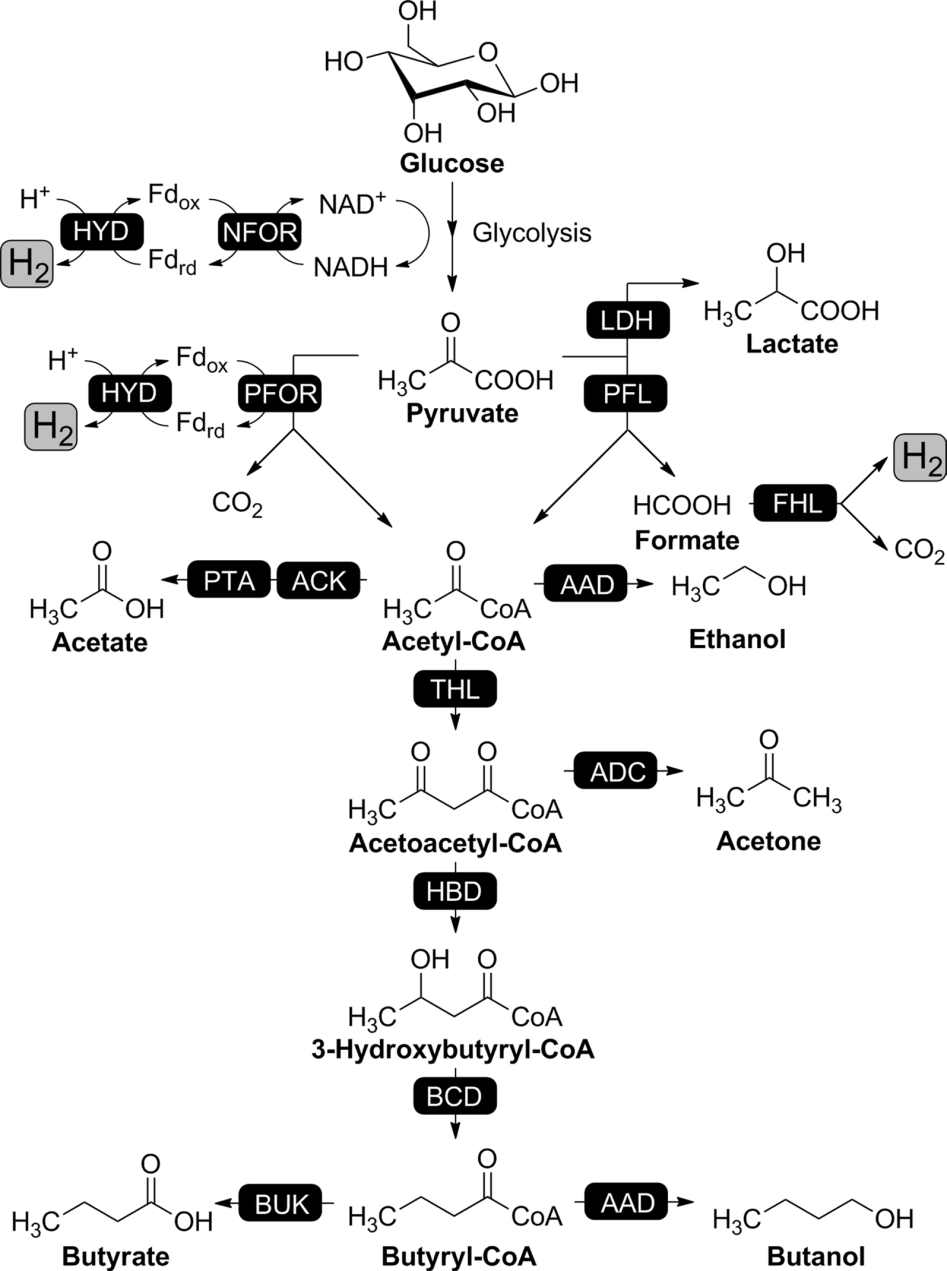
The metabolic pathways for biological hydrogen production by dark fermentation. The general substrate glucose is broken down to pyruvate through the glycolytic pathway. The fate of pyruvate differs depending on the corresponding microorganisms. The PFL pathway is employed by facultative anaerobes (shown on the right). The PFOR pathway is typical for strict anaerobes (shown on the left). Abbreviations for enzymes shown are: NFOR, NADH:ferredoxin oxidoreductase; HYD, ferredoxin-dependent hydrogenase; PFOR, pyruvate:ferredoxin oxidoreductase; ACK, acetate kinase; PTA, phosphotransacetylase; LDH, lactate dehydrogenase; PFL, formate lyase. FHL, formate:hydrogen lyase complex; AAD, aldehyde alcohol dehydrogenase; THL, thiolase; ADC, acetoacetate decarboxylase; HBD, β-hydroxybutyryl-CoA dehydrogenase; BCD, butyryl-CoA dehydrogenase; BUK, butyrate kinase

To further improve biological hydrogen production, a better understanding of the hydrogen evolution process is required. Along with the development of modern biology techniques, the molecular mechanisms for biological hydrogen production have been largely resolved. The metabolic pathways implicated in fermentative hydrogen production and the hydrogenase enzymes involved are well known and characterized in some detail. Figure 2 shows the basic metabolic pathway for biological hydrogen production by dark fermentation. At any rate, glucose or other carbon sources derived from plant biomass or waste materials will normally enter the central glycolytic pathway, generating pyruvate, ATP and NADH. Three types of key metabolic points are currently known to produce hydrogen. Pyruvate, the final product of glycolysis, is split into acetyl-CoA and formate by pyruvate: formate lyase (PFL). Formate is then cleaved into H_2_ and CO_2_ by formate: hydrogen lyase complex (FHL) [54]. This reaction is typical for the facultative anaerobes, e.g., *E. coli* [55] and Enterobacteria [56], leading to the formation of 2 H_2_/glucose.

In strict anaerobes, pyruvate is oxidatively decarboxylated under the catalysis of pyruvate: ferredoxin oxidoreductase (PFOR), along with the formation of reduced ferredoxin (Fd_rd_). The electrons in Fd_rd_ are then transferred to protons to form hydrogen by Fd-dependent hydrogenase. This is the second type of hydrogen-producing reaction which is typical for *Clostridium* species [57]. The third type of reaction utilizes NAD(P)H to evolve hydrogen. This process is catalyzed by two major enzymes, NAD(P)H: ferredoxin oxidoreductase (NFOR) and Fd-dependent hydrogenase. Fd_ox_ is reduced by NAD(P)H which is generated during catabolism and then hydrogen is formed the same as the second type reaction. This hydrogen-producing reaction is reported to exist in many thermophilic bacteria and some *Clostridium* species. Therefore, these anaerobes have a maximum theoretical yield of 4 H_2_/glucose.

Along with biological hydrogen production, ATP and NADH generated by glycolysis could also be used to produce a variety of reduced products. In facultative anaerobes, the end product is principally ethanol although some lactate is produced. In strict anaerobes, a variety of products are formed, e.g., ethanol, butyrate, butanol, acetone, depending upon the microorganisms and fermentation conditions [58].

## Screening for efficient hydrogen-producing strains

Isolation of a highly productive strain is the first step to achieve an industrial level hydrogen production. Screening for robust hydrogen-producing strains not only lays a foundation for the commercialization of fermentative hydrogen production, but also provides germplasm resources for further research on genetic modifications. A variety of efforts have been made to identify and isolate and high-yield strains for biological hydrogen production. As formate is the precursor for hydrogen in facultative anaerobes, a method for rapid determination of formate was established by a color reaction with potassium permanganate, which could be used to evaluate hydrogen production [59]. Another high-throughput screening method was established based on the coloring reagent methyl orange and the Wilkinson's catalyst. Methyl orange can be decolorized by hydrogen in the presence of Wilkinson's catalyst to indicate the hydrogen generation ability [60]. A similar screening assay to assess hydrogen production was also developed using methyl orange, methyl purple or methylene blue as the coloring agent and sulfonated iridium/rhodium cyclooctadiene triphenylphosphine as the catalyst in 96-well plates [61]. Bahona et al. [62] described a method based on fluorescence-activated cell sorting flow cytometry to screen large libraries using a hydrogen sensitive reporter module. In *E. coli*, the screenings of knock out collections of single null mutant strains (KEIO and Yale collections) have allowed finding beneficial mutations for hydrogen synthesis in a non-biased way [63, 64].

The traditional method of random mutation and screening has been very effective for isolating high-level hydrogen-producing strains despite the considerable amount of time and resources it demands. Two mutants of *Enterobacter aerogenes* were obtained by Voges-Proskauer test screening. They could produce hydrogen from glucose with yields of 1.8 mol/mol and 1.0 mol/mol, respectively [65]. Mutagenesis of *E. aerogenes* was also achieved using the atmospheric and room temperature plasma method. The best variant showed a 26.4% increase in hydrogen yield. Metabolite analysis demonstrated that the enhanced hydrogen production should attribute to the NADH pathway [66]. Ultra-violet radiation was employed for the mutagenesis of *Ethanoligenens* sp. ZGX4. Two mutants that could grow and produce hydrogen efficiently on iron-containing medium were isolated. The hydrogen evolutions of the mutants were 2356.68 ml/L and 2219.62 ml/L at a glucose concentration of 10 g/L, about 29. 71% and 22.22% higher than that of the parent strain [67]. The mutagenesis under different wavelengths of ultra-violet of the hydrogen-producing fermentation bacterium B49 was used to enhance its hydrogen production. An efficient mutant was gained and the hydrogen-producing rate was increased by 8.4% [68]. For the model microorganism *E. coli*, it was reported that chemical mutagenesis with N-methyl-N´-nitro-N-nitrosoguanidine resulted in 109-fold more hydrogen production when compared with the parental strain [69]. The hydrogen-producing marine bacterium, *Pantoea agglomerans*, was mutated by Tn7-based transposon random insertion. Hydrogen production by the best mutant strain was enhanced by 60% [70].

Although great successes have been made by the random mutagenesis and screening method, the potential shortages of these hydrogen-producing strains should be considered. One is that the mechanism for their enhanced hydrogen producing ability is not clear. It is often difficult to associate the mutants’ phenotypes to their genotypes. Recently developed tools in genome, transcriptome and proteome analysis [71] will guide the resolution of their molecular mechanisms. Based on the genome sequence, metabolomics analysis of hydrogen-producing strains could be further performed to understand metabolites changes and conduct further metabolic engineering approaches [72, 73]. Moreover, the stability of the high-producing strains should be estimated since many mutations might be the result of domestication rather than genetic variation. The trait of high hydrogen yield would be lost after several generations of serial sub-cultivation [74].

## Improving hydrogen-producing ability by rational design

Since the biosynthesis pathways for hydrogen production have been characterized in-depth, it is now possible to modify the microbial strains rationally by metabolic engineering tools. Metabolic engineering is defined as the redirection of metabolic pathways for enhanced production of existing natural products by rational design [75]. Engineering of the critical steps, altering metabolic flux to channel the flow of key intermediates and eliminating the competing pathways would lead to higher hydrogen yields. In principle, metabolic engineering requires balancing the intensity the overexpressed genes and preventing lethal mutations caused by gene knockout to maintain normal physiological metabolism of cells [76]. Many studies have been carried out on these aspects and encouraging results have been achieved during the past few years (Table 2). The rational design strategies described here would be useful in designing more robust strains for hydrogen production.

**Table 2:** Metabolic engineering strategies to improve hydrogen yields

Microorganisms	Engineering strategies	Hydrogen yields	References
*C. acetobutylicum*	Heterologous expression of hydrogenase from *C. butyricum* or overexpression of native hydrogenase	1.81 or 1.80 mol/mol glucose	[89]
*C. butyricum*	Knockout of *aad* (encoding aldehyde/alcohol dehydrogenase)	1.65 mol/mol glucose	[113]
*C. paraputrificum*	Overexpression of native [FeFe]-hydrogenase HydA	2.4 mol/mol N-acetylglucosamine	[88]
	Heterologous expression of formate dehydrogenase from *C. boidinii*	1.390 mol/mol glucose	[102]
*C. tyrobutyricum*	Knockout of *ack* (encoding acetate kinase)	0.024 g/g glucose	[114]
	Knockout of *ack* (encoding acetate kinase) and *pta* (encoding phosphate acetyltransferase)	2.61 mol/mol glucose	[115]
*E. aerogenes*	Heterologous expression of hydrogenase from *E. cloacae*	864.02 ml/g glucose	[84]
	Heterologous expression of formate dehydrogenase or FhlA (a regulator activating FHL complex) from *E. coli*	1.589 or 1.605 mol/mol glucose	[91]
	Heterologous expression of formate dehydrogenase from *C. boidinii* and knockout of *ldhA* (encoding lactate dehydrogenase)	1.702 mol/mol glucose	[101]
	Overexpression of polyphosphate kinase	1.504 mol/mol glucose	[103]
	Knockout of *nuoCDE* (encoding NADH dehydrogenase) and overexpression of nicotinic acid phosphoribosyl transferase	2.28 mol/mol glucose	[100]
*E. cloacae*	Overexpression of glutathione-S-transferase	2.55 mol/mol glucose	[96]
*E. coli*	Overexpression of native hydrogenase 3	153 mmol/mol glucose	[80]
	Engineering of the large subunit of hydrogenase 3 by directed evolution	0.84 mol/mol formate	[81]
	Heterologous expression of hydrogenase from *Synechocystis* sp. PCC 6803	1.89 mol/mol glucose	[82]
	Directed evolution of FhlA by error-prone PCR	5 μmol mg/(proteinh)	[90]
	Knockout of *hycA* (encoding a negative regulator)	31 ml/(h OD_unit_ L)	[93]
	Knockout of *hycA* and overexpression of FhlA	23.6 g/(L h)	[94]
	Knockout of *focA* (encoding formate transporter)	0.96 mol/mol glucose	[95]
	Coexpression of glucose-6-phosphate dehydrogenase and fructose-1,6-bisphosphatase	21.76 mmol/mol glucose	[98]
	Knockout of uptake hydrogenases and overexpression of the FHL complex	1.3 mol/mol glucose	[107]
	Knockout of *ldhA* and *frdBC* (encoding fumarate reductase)	1.82 mol/mol glucose	[110]
	Knockout of two uptake hydrogenases as well as *ldhA* and *frdAB* (encoding fumarate reductase)	1.80 mol/mol glucose	[111]
	Knockout of *ptsG* (encoding glucose transporter), *ldhA* and *frdD* (encoding fumarate reductase)	0.27 mol/mol glucose/xylose	[112]
*T. aotearoense*	Knockout of *ldh* (encoding lactate dehydrogenase)	2.71 mol/mol glucose	[99]

### Engineering of hydrogenases

**Fig. 3:**
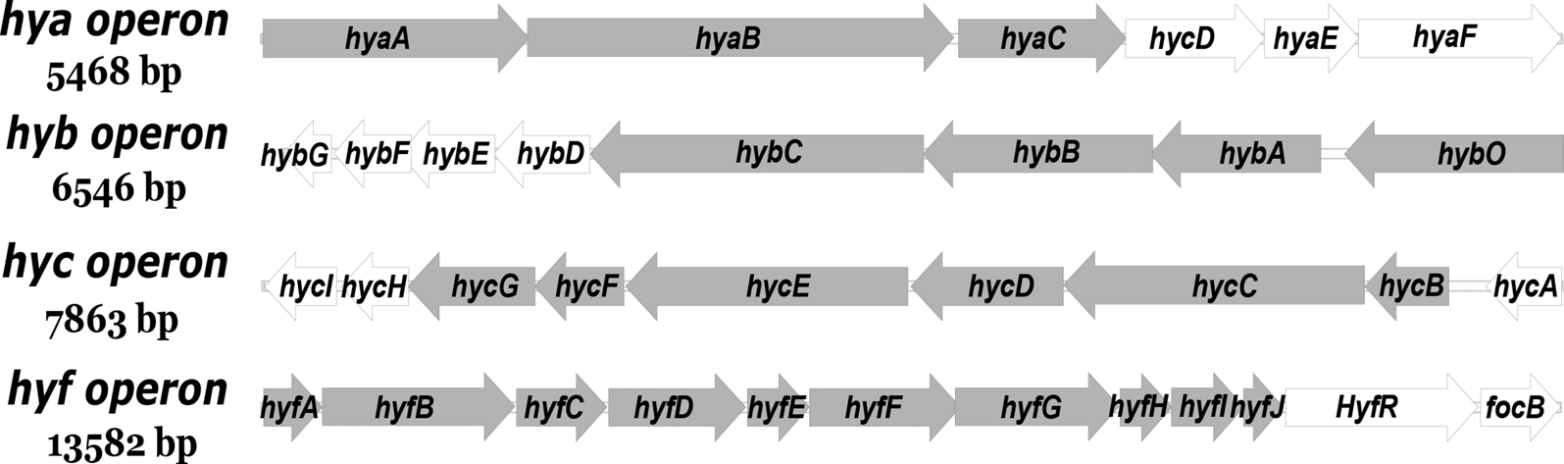
Graphic map showing the organization of the operons encoding the four hydrogenases in *E. coli*. Each hydrogenase consists of several different subunits. Arrows indicate the direction of transcription. Filled bar, structure genes; empty bar, regulatory genes

**Fig. 4:**
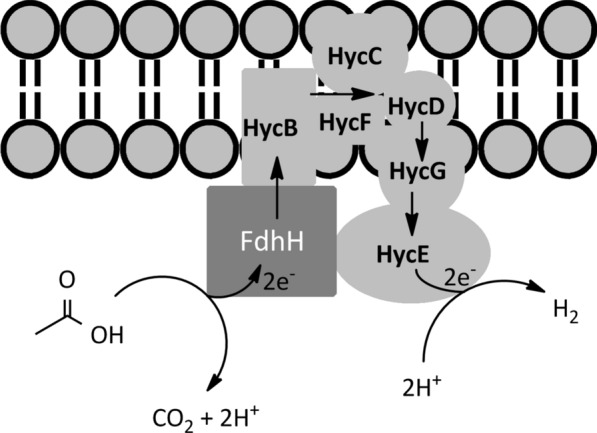
Structural models of the formate hydrogen lyase (FHL) complex in the cytoplasmic membrane of *E. coli*. The FHL complex consists of formate dehydrogenase (FdhF) and hydrogenases 3. Formate is the electron donor for FdhF, and the electron is then transferred through HycB, HycF, HycG and finally to the large subunit of hydorgenase 3 (HycE). Protons accept electrons at the large subunit to form hydrogen. Arrows indicate electron transfer through the subunits of the FHL complex

Hydrogenase, the key enzyme of the biological hydrogen production process, catalyzes the reversible reduction of H^+^ to H_2_. There are three types of hydrogenases which are evolved from independent origins: [NiFe]-hydrogenase, [FeFe]-hydrogenase and [Fe]-hydrogenase [77]. The [Fe]-hydrogenases were previously considered as "metal-free" hydrogenases until the group of Thauer showed that these enzymes actually contained a mononuclear iron active site [78]. All of the three types of hydrogenases are multimeric proteins consisting of small and large subunits, except for the case with [FeFe]-hydrogenases. Take the model microorganism *E. coli* as an example. Its genome encodes four hydrogenases (Fig. 3). Hydrogenase 1 (encoded by the *hya* operon) and hydrogenase 2 (encoded by the *hyb* operon) have hydrogen uptake activity, hydrogenase 4 (encoded by the *hyf* operon) appears to be inactive, and only hydrogenase 3 (encoded by the *hyc* operon) is a component of the FHL complex which participates in hydrogen evolution (Fig. 4) [79]. Each operon is composed of the hydrogenase structural genes and regulatory genes for their expression.

Since hydrogenases catalyze the critical step for biological hydrogen production, one strategy to enhance hydrogen productivity is to overexpress and engineer these enzymes or introduce more efficient hydrogenases in heterologous hosts. Many studies have been carried out in *E. coli*, *Clostridium* and several other hydrogen-producing bacteria. To increase the cellular hydrogenase level, the native large fragment hydrogenase 3 operon (*hycBCDEFGHI*) was cloned using the homologous recombination/plasmid rescue method and overexpressed in *E. coli*. A maximum hydrogen yield of 153 mmol/mol glucose was achieved, about 13-fold to the parental strain [80]. The large subunit (HycE) of *E. coli* hydrogenase 3 was engineered using the error-prone PCR strategy to enhance its hydrogen production ability. A mutant enzyme with 17-fold higher activity was obtained [81]. In another study, an engineered *E. coli* was constructed by heterologously expressing *Synechocystis* sp. PCC 6803 hydrogenase under the control of *alkB* promoter by a recombinant plasmid pCom10-hox. The resulting strain could produce hydrogen at a rate of 0.35 L/(L·h) while the yield of hydrogen on glucose reached 1.89 mol/mol [82]. *E. coli* native hydrogenase is sensitive to oxygen. To overcome this problem, the hydrogenase from *Hydrogenovibrio marinus* was introduced into *E. coli*. The hydrogenase was amplified by degenerate PCR based on partial sequence information in the GenBank database and cloned into pET-21b vector. In the presence of 5–10% oxygen, the recombinant strain could generate ninefold more hydrogen than the original strain [83]. Hydrogen production of *E. aerogenes* could be also increased through the introduction of a heterologous hydrogenase from *E. cloacae*. The hydrogenase gene was amplified, inserted to the prokaryotic expression vector pGEX-4 T-2 and transformed into the competent cells via electroporation to create a recombinant *E. aerogene* strain. The maximum yield reached 864.02 ml/g glucose, 1.95-fold to the wild-type strain [84]. High-level expression of the membrane-bound [NiFe]-hydrogenase in *Thermococcus kodakarensis* was achieved by using a strong constitutive promoter and the hydrogen evolution rate was enhanced by 25% [85]. Since *T. kodakaraensis* is a hyperthermophilic bacterium, it was cultured at a high temperature of 85 °C. Thermophilic hydrogen production did not require reactor cooling and restricted the risk of contamination. In addition, these producer could also endure high hydrogen concentration and had higher evolution rate [86, 87]. In the strict aerobe *Clostridium paraputrificum*, the hydA gene encoding [FeFe]-hydrogenase was identified and cloned into a shuttle vector, pJIR751, to construct a hydrogen-producing strain. 1.7-fold increase in hydrogen gas productivity was achieved when compared with the wild-type [88]. The hydrogenase from *C. butyricum* and native *C. acetobutylicum* hydrogenase were overexpressed in this host using *C. acetobutylicum*—*E. coli* shuttle vector pSOS95 including their native promoters, respectively. But hydrogen yields were comparable to the wild-type strain, which indicated that intracellular hydrogenase level was not the rate-limiting step for hydrogen production of this strain [89].

### Altering metabolic flux to the intermediates of hydrogen pathways

In order to achieve high hydrogen yield, complete oxidation of the corresponding substrates is required. The metabolic pathways for fermentative hydrogen production have been well characterized. Many studies have examined the effect of increasing metabolic flux to the hydrogen-producing pathway. The σ^54^ factor FhlA, a regulatory protein activating the expression of FHL complex in *E. coli*, was evolved by error-prone PCR. The best variant with 6 amino acid sites mutation gave a ninefold increase in hydrogen production [90]. In another study, both *fdhF* (encoding formate dehydrogenase) and *fhlA* originated from *E. coli* were coexpressed in *E. aerogenes*. The coding regions of the two genes were ligated into the pMCL plasmid, generating pMCL-fdhF and pMCL-fhlA, respectively. The recombinant plasmids were transferred into *E. aerogenes* and the resulting strain showed improved cell growth and hydrogen production rate. 38.5% enhancement of hydrogen yield was obtained [91]. The *hycA* gene of *E. coli* has been identified to encode a regulator inhibiting transcriptional activation by FhlA [92]. In an *E. coli mutant* strain knockout of *hycA*, expression of the FHL complex was up-regulated. The highest hydrogen production of this strain reached 31 ml/(h·OD_unit_·L), about twofold to the parent strain [93]. Disruption of *hycA* was further combined with *fhlA* overexpression by an low-copy-number plasmid pMW118. The transcription levels of both *fdhF* and *hycE* (encoding the large subunit of hydorgenase 3) were increased as determined by Northern blot analysis. This mutant strain was capable of producing more hydrogen than the wild-type strain [94]. In *E. coli*, formate is the direct precursor for hydrogen biosynthesis. The intracellular level of formate is critical for hydrogen production. Formate is transported out of the cell by a transporter protein, FocA. By knockout of the *focA* gene in *E. coli* W3110 using the λ-Red recombination method, the productivity and yield of hydrogen was enhanced by 52% and 78%, respectively [95]. A hydrogen-producing activator was identified and overexpressed in *E. cloacae* with the fusion-tagged protein glutathione-S-transferase. The recombinant strain could produce twofold more hydrogen as well as acetate and butyrate during hydrogen fermentation. This might be due to the metabolic flux was channeled away from by-products formation [96].

### Increasing reducing ferredoxin availability from NAD(P)H

The evolution of hydrogen from NADH is an important bifurcating pathway for biological hydrogen production. Maximizing cellular reductant flow to the hydrogenases would potentially enhance hydrogen production [97]. This assumption has been demonstrated in recent studies. The pentose phosphate pathway could produce more NADPH than glycolysis. Thus, increasing the metabolic flux through the pentose phosphate pathway would be crucial for the increase of NAD(P)H-dependent hydrogen production. It was reported that coexpression of *zwf* (encoding glucose-6-phosphate dehydrogenase) and *glpX* (encoding fructose-1,6-bisphosphatase) in *E. coli* using two compatible vectors pRSFDuet-1 and pACYCDuet-1 increased hydrogen yield by 2.32-fold [98]. The intracellular NADH could be utilized to produce ethanol, butanol, lactate, etc. Therefore, inactivation of these competitive pathways might facilitate hydrogen production. For instance, in the thermoacidophilic bacterium *Thermoanaerobacterium aotearoense*, lactate dehydrogenase was also disrupted using the homologous recombination vector pLuKELd to enhance the NADH/NAD^+^ ratio. The engineered strain showed both increased hydrogen yield and productivity [99]. To reduce the consumption of NADH, the nuoCDE genes encoding NADH dehydrogenase were disrupted using the CRISPR-Cas9 method. The hydrogen yield of the triple mutant was improved by 45.6% compared with the wild-type strain [100].

Introduction of foreign pathways to improve NADH availability would also enhance hydrogen production. The *fdh1* gene (encoding an NADH-dependent formate dehydrogenase) from *Candida boidinii* was expressed in a *ΔldhA* mutant of *E. aerogenes* which was constructed using the conjugative transfer method, to introduce an NADH regeneration system. Although the introduction of foreign *fdh1* had negative effects on the growth of the bacteria, an 86.8% increase of hydrogen yield was obtained [101]. Heterologous expression of this formate dehydrogenase in *C. paraputrificum* using the recombinant plasmid pCom10-fdh1 confirmed an improvement of the hydrogen yield at least 59% compared with the control strain [102]. In the polyphosphate kinase overexpressing *E. aerogenes* strain using the pMCL plasmid, the hydrogen production per liter of culture, the hydrogen production per cell and the hydrogen yield per mole of glucose increased by 20.1%, 12.3% and 10.8%, respectively. Metabolic flux analysis showed that the increase of the total hydrogen yield was also attributed to the improvement of NADH pathway [103].

The potential of the NADH pathway for hydrogen production is far beyond current studies. The citric acid cycle (TCA cycle), normally operational only under aerobic conditions, allows the complete oxidation of glucose to CO_2_ and NADH and FADH. If this cycle could be made to operate under anaerobic conditions, large amounts of NADH would become available. The TCA cycle is split on two branches in anaerobes [104] and facultative anaerobes upon anaerobiosis [105]. *E. coli* mutants have also been studied to find advantageous genetic backgrounds. Another problem is that the redox potential of NADH is higher than that of hydrogen. The conversion of reduced NADH to hydrogen would only be efficient if some types of reduced electron flow system are employed, which would require the input of some amount of energy.

### Eliminating the branch pathways

In the biological systems, multiple branch pathways exist to compete with the hydrogen-producing reactions. For instance, most of the hydrogen-producing bacteria also contain hydrogenases which can oxidize molecular hydrogen to protons, thus allowing bacteria to consume hydrogen. These so-called “uptake hydrogenases” are not desired for hydrogen production [106]. Thus, eliminating the genes encoding these hydrogenases would be helpful for hydrogen production and this has been carried out in practice in different microorganisms. As expected, knockout of two uptake hydrogenases Hyd1 and Hyd2 of *E. coli* by P1 transduction from the Keio library led to modest increases in hydrogen yields [107]. In another approach, hydrogen production was increased by 141-fold in an *E. coli* strain both inactivating the uptake hydrogenases successively using similar strategies and overexpressing the FHL complex [108]. Antisense RNA strategy was also used to down-regulate the expression of hydrogen uptake enzymes in *Clostridium saccharoperbutylacetonicum*, resulting in a 3.1-fold enhancement of hydrogen production [109].

Hydrogen is not the only destination for electrons in dark fermentation. In fact, fermentative bacteria transfer most of the electrons from the substrates to soluble organic acids and alcohols, such as acetate, lactate, ethanol and butanol. A big problem encountered with dark fermentation is the inhibition of hydrogen production by the accumulation of these end-products. Therefore, the genes responsible for the formation of the inhibiting products are disrupted to investigate their effects on hydrogen production. In a double mutant of *E. coli* (knock out of *ldhA* and *frdBC* encoding fumarate reductase through homologous recombination by a suicide vector), glucose metabolism was directed to the PFL complex. The hydrogen yield of this strain reached 1.82 mol/mol glucose, 1.7-fold higher than the wild-type strain [110]. In the *hycA* mutant of *E. coli*, disruption of two native uptake hydrogenases by Red recombination resulted in a hydrogen yield of 1.48 mol/mol glucose while the parental strain was 1.20 mol/mol glucose. Further deletion of *ldhA* and *frdAB* increased the hydrogen yield to 1.80 mol/mol glucose [111]. To construct an *E. coli* strain which could simultaneously utilize glucose and xylose, the *ptsG* gene was disrupted via P1 bacteriophage transduction from single-gene knockout mutant collection and 1.2-fold improvement of hydrogen production was achieved compared with the control strain. Further disruption of *ldhA* and *frdD* genes improved hydrogen yield to 0.27 mol/mol with glucose/xylose mixture or wheat straw hydrolysate as the substrates [112]. The ethanol biosynthesis pathway of *C. butyricum* was blocked through knockout of the key enzyme aldehyde-alcohol dehydrogenase (encoded by *aad*) according to the ClosTron system. The hydrogen producing capacity of this mutant increased by 20% using sodium acetate as the carbon source [113]. The acetate kinase (encoded by *ack*) of *C. tyrobutyricum* was inactivated to block the by-product acetate formation pathway using an integrational plasmid pAK-Em. The yield of hydrogen on glucose of this mutant was 0.024 g/g, a 50% enhancement of the wild-type strain [114]. The *pta* gene (encoding phosphate acetyltransferase) was further disrupted along with *ack* by transforming with non-replicative plasmids. Hydrogen yield of the double mutant increased to 2.61 mol/mol glucose with a H_2_/CO_2_ ratio of 1.43 [115].

## Conclusions and future prospects

Biological hydrogen production has been known for over 100 years and scientists applied this technology as a practical means of hydrogen fuel decades ago. It is one of the most challenging tasks in the renewable energy field in terms of fossil fuel shortage and environmental problems. Many different microbial paths for hydrogen production are under active study in recent years. The present survey reported recent effects on strain improvement for efficient hydrogen production via dark fermentation. Microorganisms employed for fermentative hydrogen production were summarized and classified. The hydrogen producing performance of various strains was introduced. The metabolic pathways and key enzymes implicated in fermentative hydrogen production were discussed in detail. Different strategies including random mutagenesis and metabolic engineering to construct an efficient hydrogen-producing strain were recommended.

Although the technical feasibility of hydrogen production by dark fermentation from renewable biomass or waste materials has been demonstrated by various researchers, the current yield and producing rate are still low. It requires us to find ideal microbial strains that can convert these raw materials efficiently to hydrogen. A literature search in this review has shown that numerous studies were applied to obtain more efficient bacterial strains. As the metabolic pathways of the major fermentative hydrogen-producing microorganisms are now well understood, it is possible to achieve a better microbial strain through rational design. However, strain improvement is just one strategy among a group of approaches to improve hydrogen yield. We cannot accomplish an industrial level of hydrogen production by this method individually, but we can easily combine it with other strategies such as fermentation engineering, bioreactor design, to finally make the microbial hydrogen-producing system economically feasible.

**Fig. 5:**
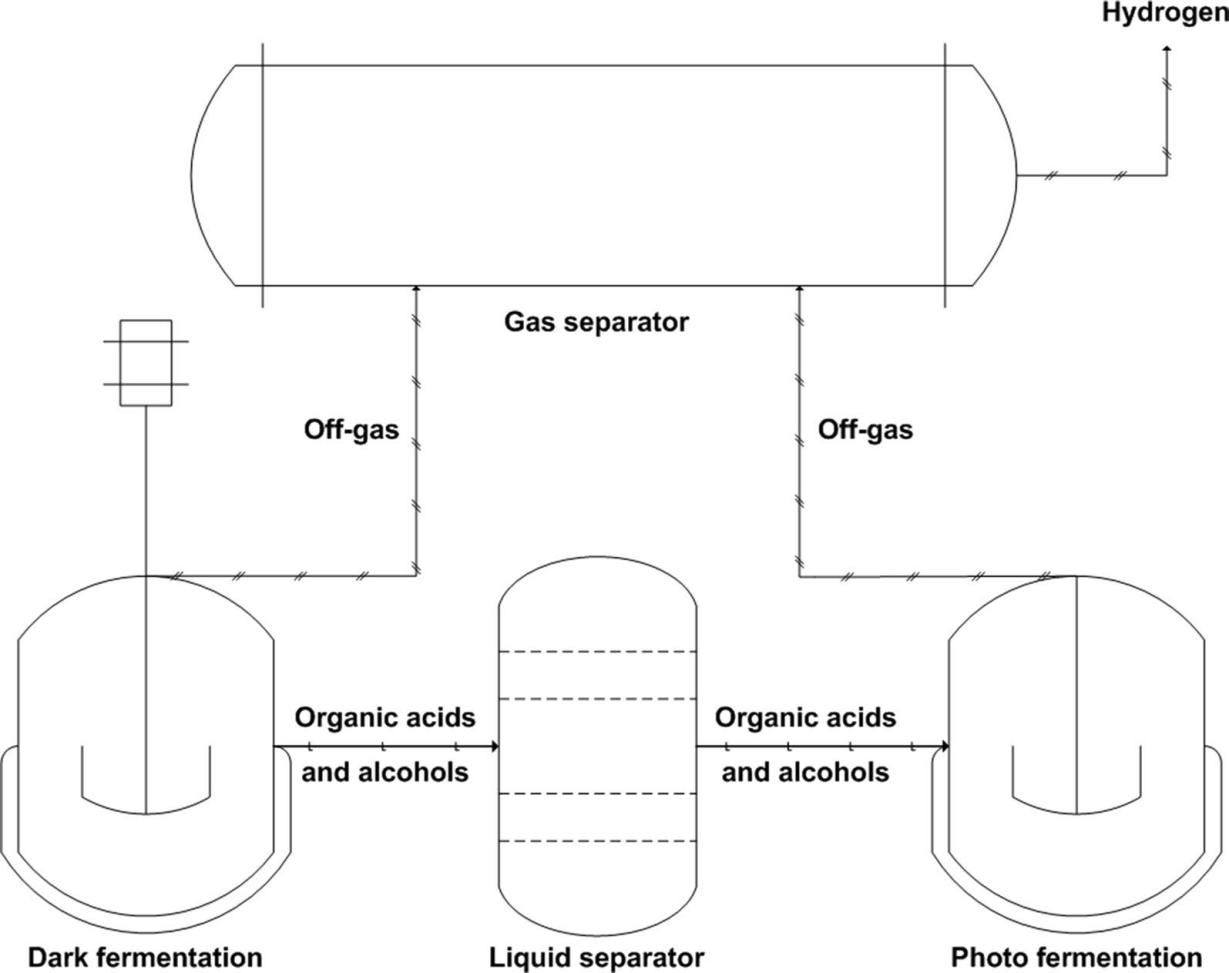
The two-step approach for hybrid fermentation by the combination of dark fermentation and photo fermentation process

The present fermentative pathways appear to place severe limits on the attainable yields with at most 33% of the potential electrons going into hydrogen production. Nevertheless, surpassing the metabolic barriers of 4 H_2_/glucose will be required since the substrates are not completely converted to hydrogen. A number of systems potentially capable of exceeding 4 H_2_/glucose have been proposed and are under active study. Hybrid fermentation process which combines dark fermentation and photo fermentation might be one of the most promising routes for the enhancement of biological hydrogen production (Fig. 5). In hybrid fermentation, the end products (organic acids and alcohols) produced by dark fermentation of carbohydrates can be further converted into hydrogen by the photosynthetic bacteria [116, 117]. Also, the biological hydrogen processes can be combined with microbial fuel cells. In microbial fuel cells, the protons move from the anode to the cathode to form hydrogen and thus have the potential to produce 8–9 H_2_/glucose [118]. In current hydrogen-producing systems, the theoretical stoichiometry of 12 H_2_/glucose (C_6_H_12_O_6_ + 12 H_2_O → 12H_2_ + 6CO_2_) is far from achieved. Despite some encouraging results, these approaches are still faced with daunting challenges that need to be addressed in future research.

Obviously, the horizon for practical application of biological hydrogen production is still in the distance. The future of biological hydrogen production depends not only on research advances, but also on economic factors, energy policies, environmental concerns and social forces. Although the search for alternative fuels is imperative and extensive research in the field of biological hydrogen production is underway, a number of technical challenges must be overcome before these technologies can be adopted on a practical large scale. The development of biological hydrogen production is a long term prospect, but we believe that industrial biological processes will become a major source of hydrogen energy in the near future.

## Data Availability

Not applicable.
